# Supplementary material for: Improved Composite Hydrogel for Bioengineered Tracheal Graft Demonstrates Effective Early Angiogenesis
Source: J Clin Med. 2024 Aug 30;13(17):5148. doi: 10.3390/jcm13175148 (PMC11396371; doi:10.3390/jcm13175148)
Supplement: Supplementary file 1 [file jcm-13-05148-s001.zip › jcm-2990520-supplementary.pdf]

Novel Composite Hydrogel for Bioengineered Tracheal Graft demonstrates Effective Early Angiogenesis

Supplement

Figure S1: Completed construct consisting of collagen-agarose hydrogel blend cast around a 3D-printed supported structure

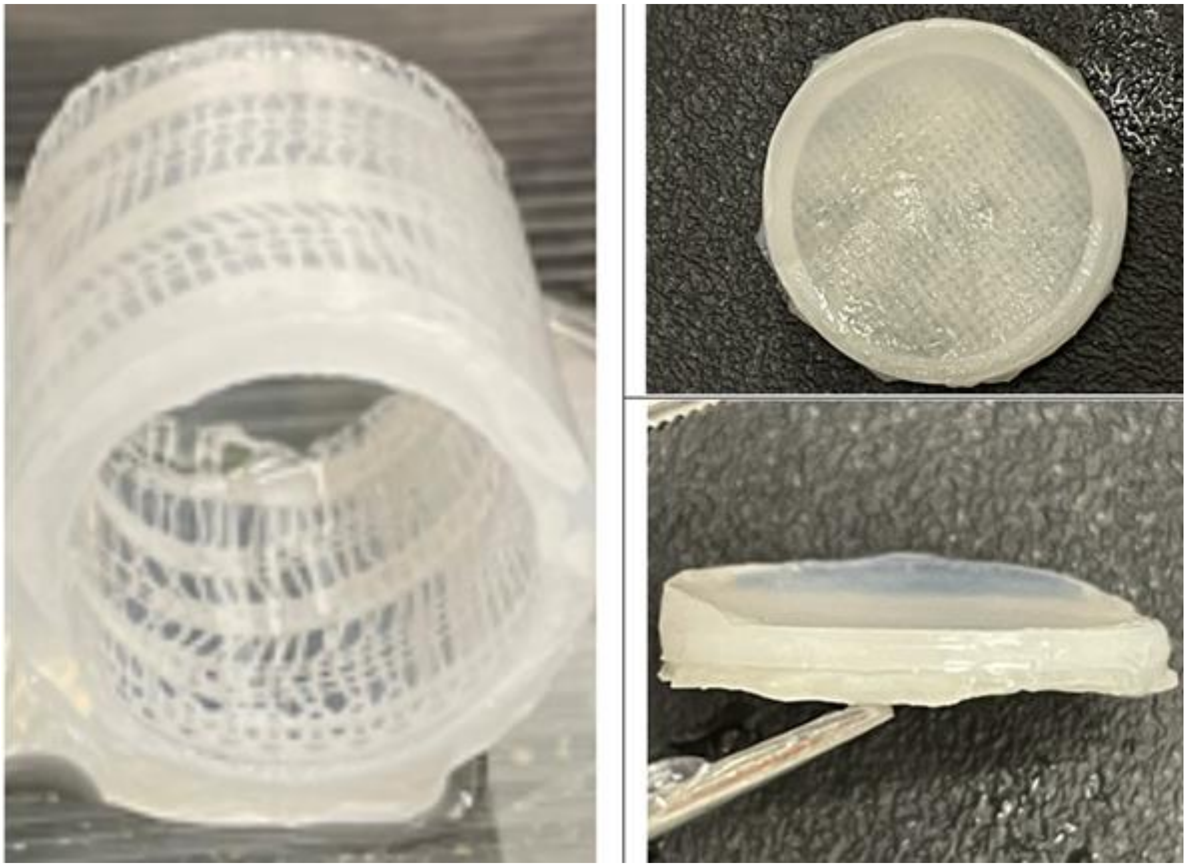

Figure S2: aSMA immunostaining of hydrogel constructs with 0.125% agarose concentration

**1mg/mL T1 Collagen 0.125% Agarose**	**2mg/mL T1 Collagen 0.125% Agarose**

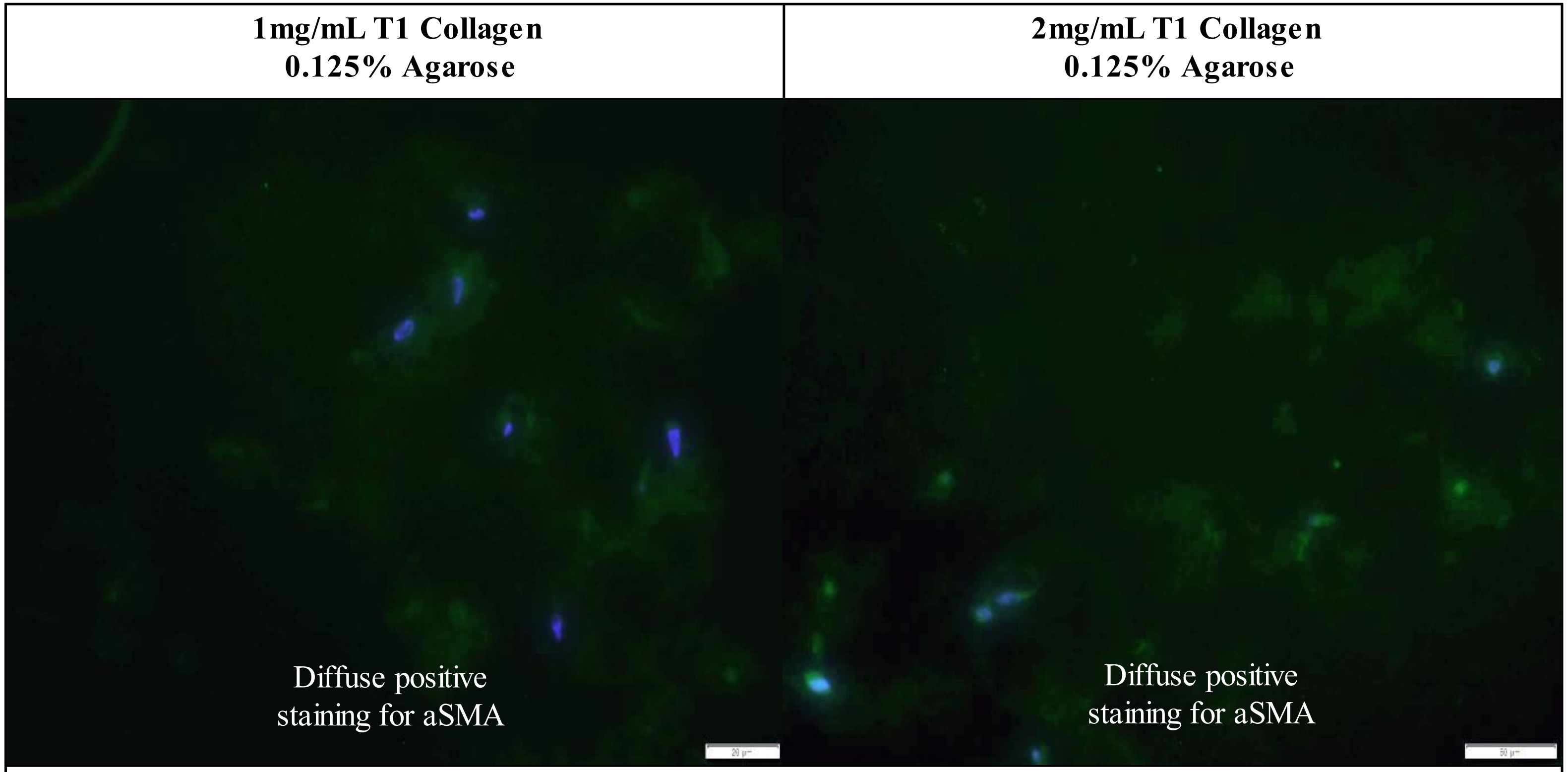

Key:
Cell Nucleus
aSMA
